# AR–PDEF pathway promotes tumour proliferation and upregulates MYC-mediated gene transcription by promoting MAD1 degradation in ER-negative breast cancer

**DOI:** 10.1186/s12943-018-0883-0

**Published:** 2018-09-14

**Authors:** Lu Cao, Cong Xu, Guomin Xiang, Fang Liu, Xiaozhen Liu, Congying Li, Jing Liu, Qingxiang Meng, Jiao Jiao, Yun Niu

**Affiliations:** Department of Breast Cancer Pathology and Research Laboratory, Key Laboratory of Breast Cancer Prevention and Therapy, Tianjin Medical University Cancer Institute and Hospital, National Clinical Research Center for Cancer, Key Laboratory of Cancer Prevention and Therapy, Tianjin’s Clinical Research Center for Cancer, Tianjin Medical University, Ministry of Education, Tianjin, 300060 China

**Keywords:** Androgen receptor, Prostate-derived Ets factor, MYC, MAD1, Breast cancer

## Abstract

**Background:**

Androgen receptor (AR) is expressed in 60%~ 70% oestrogen receptor (ER)-negative breast cancer (BC) cases and promotes the growth of this cancer subtype. Expression of prostate-derived Ets factor (PDEF), a transcription factor, is highly restricted to epithelial cells in hormone-regulated tissues. MYC and its negative regulator MAD1 play an important role in BC progression. Previously, we found that PDEF expression is strongly correlated with AR expression. However, the relationship between AR and PDEF and the function of PDEF in ER-negative BC proliferation are unclear.

**Methods:**

AR and PDEF expression in ER-negative BC tissues and cell lines was determined by performing immunohistochemistry or western blotting. Protein expression levels and location were analysed by performing western blotting, RT-qPCR and immunofluorescence staining. Co-immunoprecipitation and chromatin immunoprecipitation assays were performed to validate the regulation of AR–PDEF–MAD1–MYC axis. Moreover, the effect of AR and PDEF on BC progression was investigated both in vitro and in vivo.

**Results:**

We found that PDEF was overexpressed in ER-negative BC tissues and cell lines and appeared to function as an oncogene. PDEF expression levels were strongly correlated with AR expression in ER-negative BC, and *PDEF* transcription was positively regulated by AR. PDEF upregulated MYC-mediated gene transcription by promoting MAD1 degradation in ER-negative BC. Finally, we found that compared with the inhibition of AR expression alone, simultaneous inhibition of AR and PDEF expression further suppressed tumour proliferation both in vitro and in vivo.

**Conclusions:**

Our data highlight the role of the AR–PDEF–MAD1–MYC axis in BC progression and suggest that PDEF can be used as a new clinical therapeutic target for treating ER-negative BC.

**Electronic supplementary material:**

The online version of this article (10.1186/s12943-018-0883-0) contains supplementary material, which is available to authorized users.

## Background

Endocrine therapies for breast cancer (BC) that target oestrogen receptor (ER) are ineffective in 25%~35% cases of ER-negative BC [1]. Studies have detected high androgen receptor (AR) expression levels in 60%~70% ER-negative BC cases, thus highlighting the importance of AR in the biology of this cancer subtype [2, 3]. AR is critical for promoting the growth and malignancy of ER-negative BC, and AR targeting is a potential therapeutic strategy for treating some patients with ER-negative BC [4]. Although some mechanisms underlying this oncogenic role of AR in ER-negative BC have been identified, it is important to identify other pathways for designing additional therapies for treating patients with ER-negative BC.

Prostate-derived Ets factor (PDEF) is a transcription factor belonging to Ets transcription factor family. Ets transcription factors are highly conserved proteins with a unique 85-amino-acid DNA-binding domain and recognise a core 5′-GGAA/T-3′ sequence present in downstream target genes [5, 6]. PDEF was first identified as a co-regulator of AR and an activator of prostate-specific antigen [7]. Moreover, PDEF expression is highly restricted to epithelial cells present in hormone-regulated tissues such as prostate gland, breast and ovaries [8, 9]. PDEF regulates tumour growth, and loss of PDEF expression is associated with a highly aggressive phenotype of prostate and colon cancers [10, 11]. However, it is unclear whether PDEF functions similarly in breast carcinoma. Several studies have shown that PDEF expression is downregulated in invasive basal BC cell lines and that PDEF re-expression inhibits BC cell proliferation and migration, suggesting that it plays a tumour-suppressive role [12]. In contrast, PDEF expression is enriched in luminal tumours and is correlated with poor overall survival (OS) of patients with ER-positive BC, suggesting that it has an oncogenic function [13]. Recent global gene expression studies have shown that high *PDEF* expression is often associated with AR positivity in ER-negative BC [14]. We previously observed that PDEF was overexpressed in ER-negative BC and that its expression was strongly correlated with AR expression; moreover, our results suggested that *PDEF* may be a downstream target gene of AR and a potential prognostic factor [15].

MYC expression promotes BC proliferation and malignancy [4, 16, 17]. MYC–MAX–MAD network is important for regulating cell physiology [18, 19]. This network includes transcriptional regulators that form different heterodimers that activate or repress target gene expression. Thus, the proteins in this network function as a molecular switch to regulate gene expression. MYC together with its heterodimerisation partner MAX functions as a tumour-promoting transcriptional regulator [17, 19]. In contrast, MAD1, a member of this network, functions as a transcriptional repressor and interacts with MAX to deactivate this molecular switch, thus antagonising the MYC–MAX complex that activates this molecular switch [20].

In the present study, we investigated the role of PDEF and its relationship with AR in ER-negative BC. Our results showed that PDEF was overexpressed in ER-negative BC and acted as an oncogene. PDEF levels were strongly correlated with AR expression in ER-negative BC, and *PDEF* transcription was positively regulated by AR. Moreover, we found that PDEF upregulated MYC-mediated gene transcription by promoting MAD1 degradation in ER-negative BC. Thus, our results suggest that PDEF is a clinically useful target for treating patients with ER-negative BC and highlight a novel mechanism of the AR signalling pathway in ER-negative BC proliferation.

## Methods

### Clinical specimens

In all, 100 ER-negative invasive BC specimens and their corresponding adjacent normal tissues were collected from the Cancer Hospital of Tianjin Medical University from 1 January to 31 December 2008. All resources were characterised and included patients’ clinical and pathological data. None of the patients received any preoperative treatment. Samples for western blotting were randomly selected from these 100 specimens (*N* = 8). Study protocols were reviewed and approved by the Institutional Ethics Committee of Tianjin Medical University Institute and Cancer Hospital. OS was defined as the time (in months) from the last follow-up visit or the interval between tumour resection and death due to BC. Disease-free survival (DFS) was defined as the interval (in months) between surgery for a confirmed local relapse or distant recurrence. All the 100 cases were investigated and followed up from 108 to 120 months until 31 December 2017.

### Cell culture conditions and treatments

BC cell lines MDA-MB-453 and SKBR-3 used in this study were purchased from Type Culture Collection of the Chinese Academy of Sciences, Shanghai, China. Results of gene profiling studies have shown that MDA-MB-453 cells are molecular apocrine (ER^−^/PR^−/^AR^+^) BC cells and show high AR expression [14]. MDA-MB-453 cells were cultured in L15 medium (Gibco, USA) containing 10% foetal bovine serum (FBS; Gibco) and 1% penicillin/streptomycin (Life Technologies, USA) at 37 °C in an incubator lacking CO_2_. SKBR-3 cells were cultured in RPMI 1640 medium supplemented with 10% FBS at 37 °C in a 5% CO_2_ incubator. Next, the two cell lines were treated with 1 nM dihydrotestosterone (DHT; Sigma-Aldrich, USA) for 0 or 48 h or with different dose of DHT for 48 h.

### Immunohistochemistry

Immunohistochemistry (IHC) analyses were performed as described previously [21]. Antibodies against AR (ab9474; dilution, 1:200), PDEF (ab197375; dilution, 1:200), MAD1 (ab175245; dilution, 1:200) and MYC (ab32072; dilution, 1:200) were purchased from Abcam. Anti-Ki67 antibody (sc-23,900; dilution, 1:200) was purchased from Santa Cruz Biotechnology. Normal breast tissue sections were processed simultaneously and were used as positive controls for AR and PDEF, and normal goat serum-substituted primary antibodies were used as negative controls. Two senior pathologists independently quantified IHC slides. IHC scores of PDEF were used to the multiplied result of percentage positivity and staining intensity in the stained tissue area, and total scores ranged from 0 to 6. Percentage positivity was scored as 0 (0–25%), 1 (26–50%) and 2 (> 50%), and staining intensity was scored as 0 (no staining), 1 (weak staining), 2 (moderate staining) and 3 (strong staining). A total score of ≥0 and ≤ 3 indicated negative PDEF expression, and a total score of ≥4 indicated positive PDEF expression [21]. AR expression was considered to be positive if nuclear staining was observed in > 10% tumour cells.

### Western blotting

Western blotting was performed as described previously [21] by using the following primary antibodies: anti-AR antibody (ab9474; dilution, 1:3000), anti-PDEF antibody (ab53881; dilution, 1:1000; Abcam), anti-MAD1 antibody (ab175245; dilution, 1:3000), anti-MYC antibody (ab32072; dilution, 1:3000), anti-β-catenin antibody (ab32572; dilution, 1:3000; Abcam), anti-AKT antibody (sc-135,829; dilution, 1:3000; Santa Cruz Biotechnology), anti-phosphorylated AKT antibody (anti-p-AKT; sc-7985-R; dilution, 1:3000; Santa Cruz Biotechnology), anti-ERK antibody (sc-514,302; dilution, 1:3000; Santa Cruz Biotechnology), anti-phosphorylated ERK antibody (anti-p-ERK; sc-81,492; dilution, 1:3000; Santa Cruz Biotechnology) and anti-EGFR antibody (ab52894; dilution, 1:3000; Abcam).

### Immunofluorescence staining

Immunofluorescence staining was performed as described previously [21]. For this, BC tissue sections or cells were stained with the antibodies against AR (ab9474; dilution, 1:200) and PDEF (ab53881; dilution, 1:200). Quantification was performed using 4–6 independent fields.

### Quantitative reverse transcription-PCR

Quantitative reverse transcription-PCR (RT-qPCR) was performed using a standard protocol given in SYBR Green PCR kit (Toyobo, Osaka, Japan) and by using iQ5 quantitative PCR system (Bio-Rad, USA). Ct values of each gene obtained from triplicate reactions were averaged. Target gene expression was quantified by normalising the average Ct value of the target gene to that of housekeeping gene *GAPDH* (ΔCt) and was expressed as 2-ΔCt. Primers used for performing qPCR are listed in supplemental document.

### Lentiviral infection

Lentivirus infection was performed using Lenti-Pac™ HIV Expression Packaging Kit (GeneCopoeia, Guangzhou, China). Lentiviruses produced in 293 T cells were used to infect BC cells cultured in a medium containing 5 μg/mL polybrene. Lentiviral vectors expressing four independent shRNAs against PDEF or AR and those inducing PDEF or MAD1 overexpression were obtained from GeneCopoeia. After the infection, cells were selected using puromycin.

### Lentiviral infection and shRNA transfection

For transfection, BC cells were seeded in an antibiotic-deficient complete medium one day before the experiment. After 24 h, the cells were transfected with 50 nM shRNA by using Lipofectamine 2000 (Invitrogen). At 48 h after the transfection, the cells were harvested and analysed by performing RT-qPCR and western blotting. We used *PDEF*-shRNA no. #2 for lentiviral preparation. Lentivirus infection was performed using the Lenti-Pac™ HIV Expression Packaging Kit. Lentiviruses produced in 293 T cells were used to infect BC cells cultured in the medium containing 5 μg/mL polybrene, and infected cells were selected using puromycin. The shRNAs used in this study are listed in supplemental document.

### Co-immunoprecipitation assay

Cell lysates were generated using Complete Mini protease inhibitor cocktail (Roche Diagnostics, Mannheim, Germany). Total protein concentration in the cell lysates was measured using Pierce BCA protein assay kit (Thermo Scientific, Bonn, Germany) and was analysed using Eppendorf Master Photometer. Co-immunoprecipitation (Co-IP) assay was performed using the cell protein lysates and Pierce Co-IP kit (Thermo Scientific), according to the manufacturer’s protocol. For this, 10 μg anti-AR antibody (ab9474) or anti-PDEF antibody (ab53881) was incubated with a delivered resin and was covalently coupled. The antibody-coupled resin was incubated with the cell protein lysates overnight at 4 °C. Next, the resin was washed, and protein complexes bound to the antibody were eluted and examined by performing western blotting.

### Chromatin immunoprecipitation assay

Chromatin immunoprecipitation (ChIP) assay was performed according to manufacturer’s (Millipore) instructions. Briefly, DNA in BC cell was cross-linked with histones by adding formaldehyde for 10 min at room temperature. BC cell were sonicated in SDS lysis buffer to produce cell lysates containing 300- to 500-bp chromatin fragments. Next, antibodies were incubated with Dynabead proteins A and G (Invitrogen) for 6 h, followed by overnight incubation with the sonicated cell lysates for chromatin collection. Amount of immunoprecipitated DNA was normalised to that in the input and was expressed relative to the amount of DNA present in a negative control intergenic region. Primers used for the ChIP assay are listed in supplemental document.

### Transwell and wound-healing assays

The upper chamber of a Transwell was coated with Matrigel (BD Bioscience, USA) for performing cell invasion assay. Briefly, BC cells (density, 1 × 10^5^ cells) were seeded and incubated in the upper chamber containing an FBS-deficient medium. The lower chamber was filled with a 10% FBS-containing medium. After incubation at 37 °C for 24 h, the cells in the upper chamber were removed with a cotton swab. The reverse face of the membrane contained cells that had invaded the membrane. The invaded cells were fixed with 4% paraformaldehyde and stained with Giemsa. Cell migration was assessed by performing wound-healing assay. For this, BC cells (density, 1 × 10^6^ cells) were cultured in a 3-cm dish and were wounded using a 100 μL plastic pipette tip. After 48 h, the size of the wound was measured and photographed.

### CCK-8 cell proliferation assay

Cell proliferation assay was performed using Cell Counting Kit-8 (Dojindo, Japan). Briefly, BC cells were plated in 96-well plates in triplicate at an approximate density of 3 × 10^4^ to 5 × 10^4^ cells per well and were cultured under a standard culture condition. The cells were then treated with the indicated reagent, and the number of cells per well was determined by measuring absorbance (450 nm) at indicated time points.

### Flow cytometry analysis

Cell cycle analysis was performed by staining BC cells with PI by using CycleTEST™ PLUS DNA reagent kit (BD Biosciences), according to the manufacturer’s instruction.

### Colony formation assay

Approximately 500 BC cells were seeded in each well of a six-well plate and were incubated for 7 days. Colonies of these cells were fixed with methanol for 30 min and were stained with 0.1% crystal violet for 1 h.

### Xenograft

Treated BC cells (density, 3 × 10^6^ cells) together with 100 μg Matrigel were inoculated into the mammary fat pads of 5-week-old female SCID mice. Tumour growth was recorded twice a week with a caliper-like instrument. Tumour volume was calculated using the formula tumour volume = (width^2^ × length)/2. The mice were sacrificed after 6 weeks according to the guidelines for the welfare and use of animals in cancer research, and the final tumour volume and weight were determined. All in vivo experiments were reviewed and approved by the Animal Ethics Committee of TMUCIH and were performed according to the guidelines for the welfare and use of animals in cancer research and national law [22].

### H&E staining

Tissues were fixed in 10% neutral-buffered formalin for 24 h, embedded in paraffin, cut into 4-μm-thick sections, deparaffinised with xylene and processed with a graded ethanol series. Next, the sections were stained with H&E and were observed using BX51 microscope (Olympus).

### Statistical analysis

Data are presented as mean ± standard deviation (SD) of at least three independent experiments. Student’s *t*-test, χ^2^ test and Fisher’s exact test were used to compare two groups by using SPSS 22.0 (IBM, Chicago, IL, USA). Kaplan–Meier test was used to estimate the OS and RFS. *p* < 0.05 was considered statistically significant.

## Results

### PDEF co-expresses with AR in ER-negative BC tissues

**Fig. 1:**
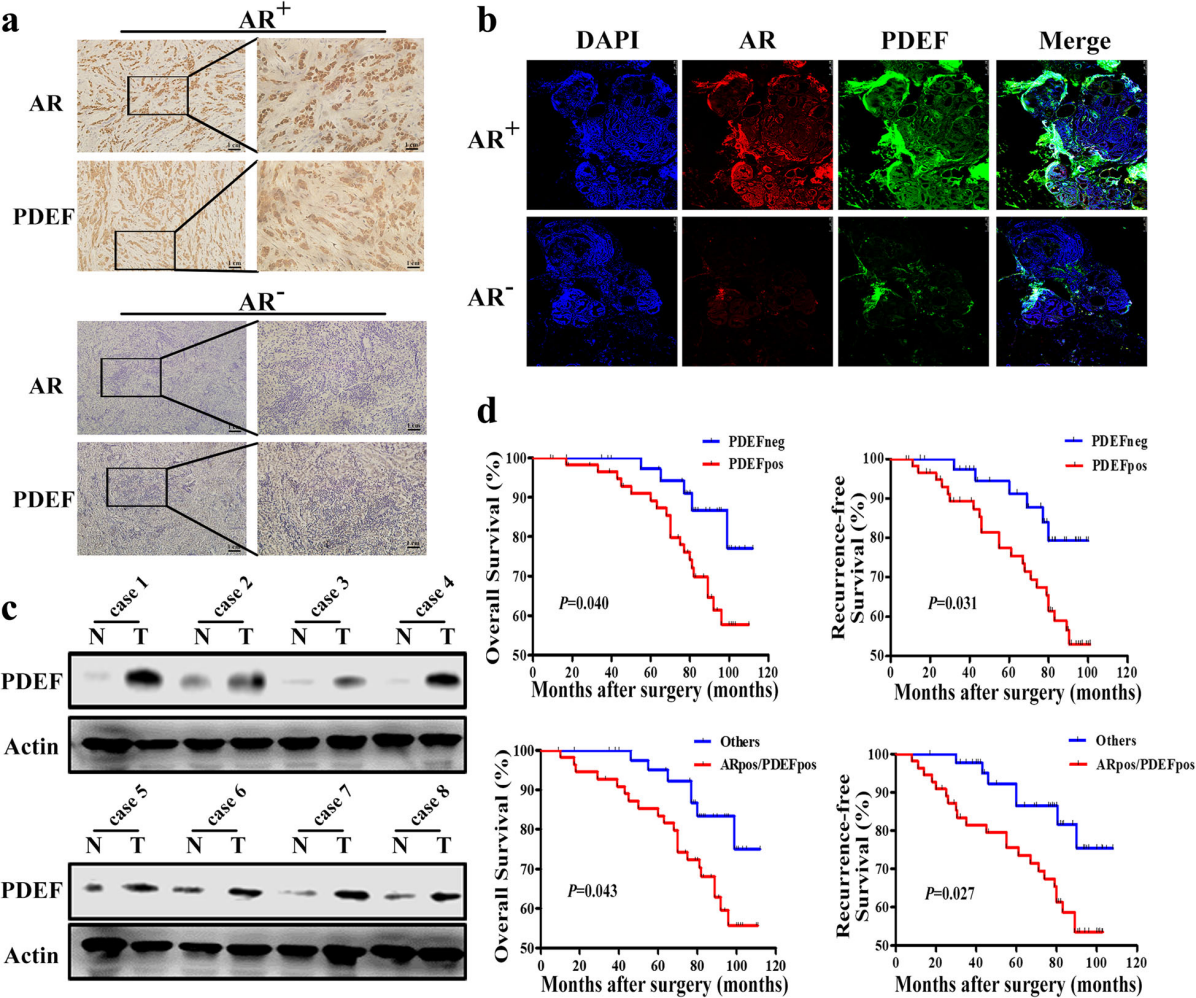
PDEF co-expresses with AR in ER-negative BC tissues. **a** IHC staining showing the co-expression of AR and PDEF in an AR^+^ BC specimen and no expression of both AR and PDEF in an AR^−^ BC specimen (magnification, × 100 and × 400). **b** Immunofluorescence staining showing the co-expression of AR and PDEF in an AR^+^ BC specimen and no expression of both AR and PDEF in an AR^−^ BC specimen (magnification, × 100). **c** PDEF protein expression levels in BC tissues (T) and paired non-tumour tissues (N) were examined by performing western blotting. The non-tumour tissues corresponded to adjacent normal tissues in the same patient. **d** Kaplan–Meier curves showing the OS (top left, *P* = 0.040) or RFS (top right, *P* = 0.031) of patients with PDEF^+^ vs. PDEF^−^ tumours. Kaplan–Meier curves showing the OS (bottom left, *P* = 0.043) or RFS (bottom right, *P* = 0.027) of patients with AR^+^PDEF^+^ vs. others tumours

AR and PDEF expression levels were first examined by performing IHC analysis of the 100 ER-negative BC tissues. PDEF showed a nuclear staining pattern, with little or no cytoplasmic or membranous staining. Of the 100 samples, 60 (60%) showed positive nuclear PDEF expression (PDEF^+^) and 69 (69%) showed positive nuclear AR expression (AR^+^). PDEF expression was associated with tumour grade (*p* = 0.032), pTNM stage (*P* = 0.011), lymphatic metastasis (*P* < 0.001) and AR expression (*P* < 0.001) (Table 1). PDEF^+^ tumours significantly more often showed AR positivity (Fig. 1a and b). AR and PDEF were more often co-expressed, with 55 (55%) cases having AR^+^PDEF^+^ tumours. Next, we examined the clinicopathological variables between AR^+^PDEF^+^ and others in the 100 ER-negative BC specimens and found that AR^+^PDEF^+^ was associated with pTNM stage (*P* = 0.047) and HER2 expression (*P* = 0.039) (Table 1). Results of western blotting showed that PDEF protein expression was higher in ER-negative BC tissues than the corresponding adjacent normal tissues (Fig. 1c). Kaplan–Meier survival analysis showed that high PDEF expression was associated with poor OS (*P* = 0.040) and RFS (*P* = 0.031); moreover, AR and PDEF co-expression was associated with poor OS (*P* = 0.043) and RFS (*P* = 0.027) (Fig. 1d).

**Table 1:** The Relationship of PDEF expression alone and AR and PDEF co-expression with various clinicopathological parameters and other biomarkers in the 100 ER-negative breast cancer cases

Variable	PDEF expression (%)		*P* value	AR/PDEF expression (%)		*P* value
	Negative 40 (40.0)	Positive 60 (60.0)		AR^+^PDEF^+^ 55 (55.5)	Others 45 (45.0)	
Age						
≤ 49	19 (36.5)	33 (63.5)	0.298	30 (57.7)	22 (42.3)	0.359
> 49	21 (43.8)	27 (56.2)		25 (52.1)	23 (47.9)	
Tumor size						
< 2 cm	12 (44.4)	15 (55.6)	0.841	14 (51.9)	13 (48.1)	0.803
2~ 5 cm	18 (37.5)	30 (62.5)		29 (60.4)	19 (39.6)	
> 5 cm	10 (40.0)	15 (60.0)		12 (48.0)	13 (52.0)	
Menopausal status						
No	18 (42.9)	24 (57.1)	0.385	22 (52.4)	20 (47.6)	0.403
Positive	22 (37.9)	36 (62.1)		33 (56.9)	25 (43.1)	
Grade						
1	5 (71.4)	2 (28.6)	0.032^a^	2 (28.6)	5 (71.4)	0.053
2	17 (45.9)	20 (54.1)		18 (48.6)	19 (51.4)	
3	18 (32.1)	38 (67.9)		35 (62.5)	21 (37.5)	
pTNM stage						
TNM I	12 (75.0)	4 (25.0)	0.011^a^	4 (25.0)	12 (75.0)	0.047^a^
TNM II	21 (34.4)	40 (65.6)		37 (60.7)	24 (39.3)	
TNM III	7 (30.4)	16 (69.6)		14 (60.9)	9 (39.1)	
Lymphatic metastasis						
No	34 (61.8)	21 (38.2)	< 0.001^a^	22 (52.4)	20 (47.6)	0.403
Positive	6 (13.3)	39 (86.7)		33 (56.9)	25 (43.1)	
AR						
Negative	26 (83.9)	5 (16.1)	< 0.001^a^	–	–	–
Positive	14 (20.3)	55 (79.7)		–	–	
PR						
Negative	36 (41.4)	51 (58.6)	0.341	46 (52.9)	41 (47.1)	0.211
Positive	4 (30.8)	9 (69.2)		9 (69.2)	4 (30.8)	
HER2						
Negative	8 (26.7)	22 (73.3)	0.058	21 (70.0)	9 (30.0)	0.039^a^
Positive	32 (45.7)	38 (54.3)		34 (48.6)	36 (51.4)	
Ki-67						
< 20%	7 (31.8)	15 (68.2)	0.263	14 (63.6)	8 (36.4)	0.250
≥ 20%	33 (42.3)	45 (57.7)		41 (52.6)	37 (47.4)	
P53						
Negative	17 (33.3)	34 (66.7)	0.118	31 (60.8)	20 (39.2)	0.162
Positive	23 (46.9)	26 (53.1)		24 (49.0)	25 (51.0)	
VEGF						
Negative	12 (54.5)	10 (45.5)	0.092	10 (45.5)	12 (54.5)	0.218
Positive	28 (35.9)	50 (64.1)		45 (57.7)	33 (42.3)	

^a^Significantly different

### PDEF is directly regulated by AR

**Fig. 2:**
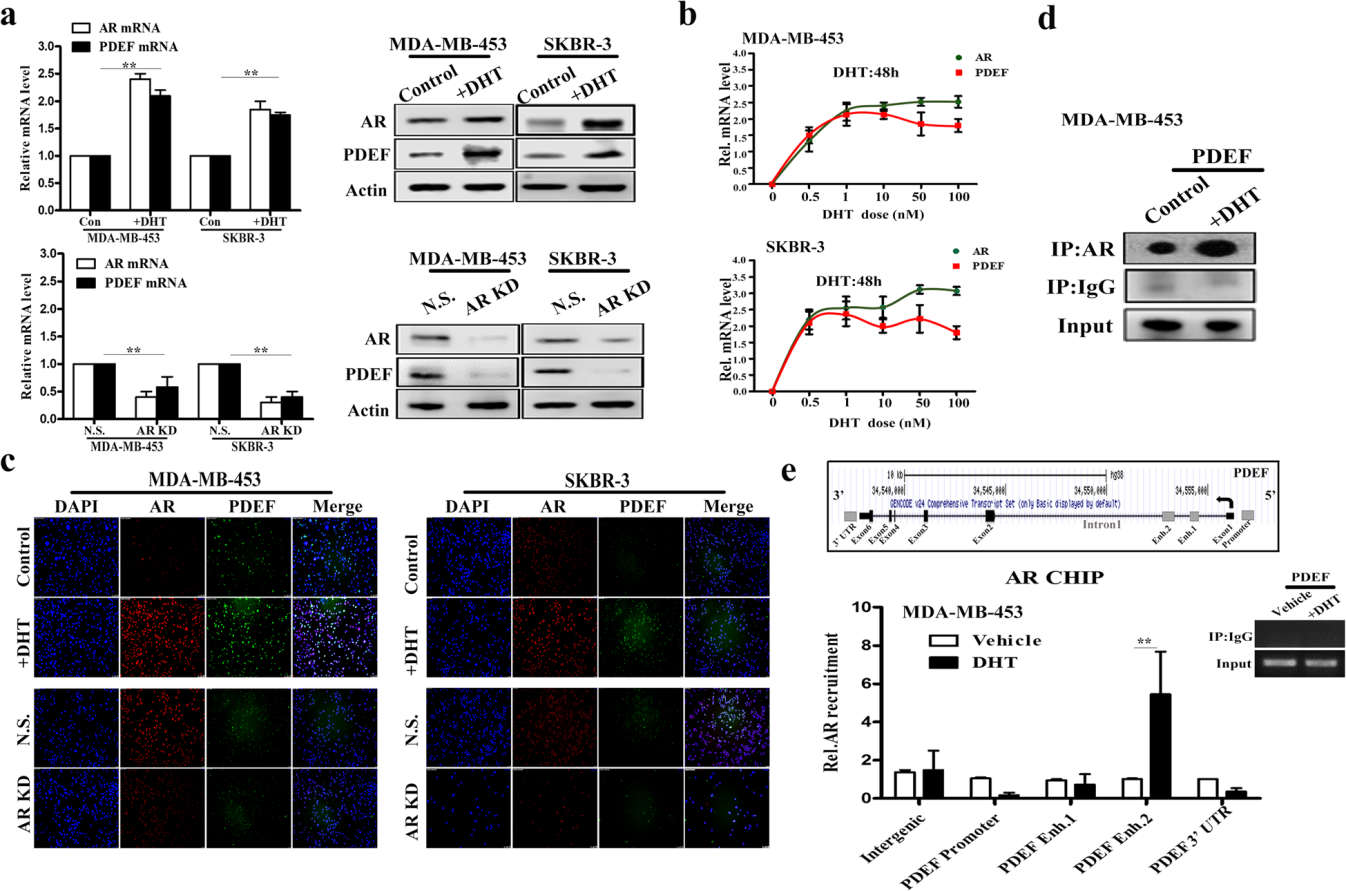
PDEF is directly regulated by AR**. a** *AR* and *PDEF* mRNA and protein levels were determined by performing RT-qPCR (top left panels) and western blotting (top right panels), respectively, of MDA-MB-453 and SKBR-3 cells treated with 1 nM DHT for 48 h (+DHT) or without DHT (control). *AR* and *PDEF* mRNA and protein levels were determined by performing RT-qPCR (bottom left panels) or western blotting (bottom right panels), respectively, of MDA-MB-453 and SKBR-3 cells infected with a non-specific (NS) shRNA- or AR-shRNA-expressing lentiviral vector (KD: knockdown). Data are presented as mean with SD. **b** *AR* and *PDEF* mRNA levels were determined by performing RT-qPCR of MDA-MB-453 (above) and SKBR-3 (under) cells treated with increasing DHT doses for 48 h. The mRNA levels are presented as mean with SD and have been normalised using those of the housekeeping gene *GAPDH*. **c** AR and PDEF protein levels were determined by performing immunofluorescence staining of MDA-MB-453 and SKBR-3 cells treated with 1 nM DHT for 0 h (control) or 48 h (+DHT). AR and PDEF protein levels were determined by performing immunofluorescence staining of MDA-MB-453 and SKBR-3 cells infected with an NS shRNA- or AR-shRNA-expressing lentiviral vector (KD: knockdown). **d** Co-IP assay was performed with an anti-AR antibody in MDA-MB-453 cells treated with 1 nM DHT for 48 h and control vector-infected cells. The interaction between precipitated PDEF and AR was detected using the anti-AR antibody. **e** Top panel: Schematic diagram of the AR-binding regions within the *PDEF* locus. Enh: enhancer. Lower left panel: Results of the direct AR ChIP assay followed by RT-qPCR of MDA-MB-453 cells treated with vehicle (white bars) or 1 nM DHT (48 h, black bars); data are presented as mean ± SD. Lower right panel: Semi-quantitative PCR of a negative control (IgG) sample

ER-negative and AR-positive BC cell lines MDA-MB-453 [14, 23] and SKBR-3 [24] were treated with 1 nM DHT for 48 h to promote AR expression or were infected with an AR-shRNA-expressing lentiviral vector to inhibit AR expression. Upregulated AR expression promoted *PDEF* mRNA and protein overexpression, whereas downregulated AR expression significantly inhibited *PDEF* mRNA and protein expression (Fig. 2a). Next, we performed immunofluorescence staining to reassess AR and PDEF protein levels and obtained the same results (Fig. 2c). A time-course analysis of MDA-MB-453 and SKBR-3 cells showed that treatment with increasing DHT doses strongly increased *PDEF* mRNA expression (Fig. 2b). These results indicated a positive role of AR in the regulation of PDEF expression. We next examined whether AR protein physically interacted with PDEF protein. We found that AR co-immunoprecipitated with PDEF in both MDA-MB-453 (Fig. 2d) and SKBR-3 cells (Additional file 1: Figure S1a) because of DHT-induced interaction between these proteins. To further examine whether AR regulated PDEF expression, we examined four AR-binding regions in the *PDEF* locus mentioned in a previously established AR cistrome dataset [25]. The first one is located within the *PDEF* promoter, two enhancers (1 and 2) are located within the first intron of *PDEF* and the fourth one is located within the 3′-untranslated region of *PDEF*. Direct AR ChIP assay by using MDA-MB-453 cells showed DHT-induced recruitment of AR at the second enhancer of *PDEF*, thus confirming that *PDEF* was a direct target of AR (Fig. 2e).

### PDEF promotes the proliferation of ER-negative BC cell lines

**Fig. 3:**
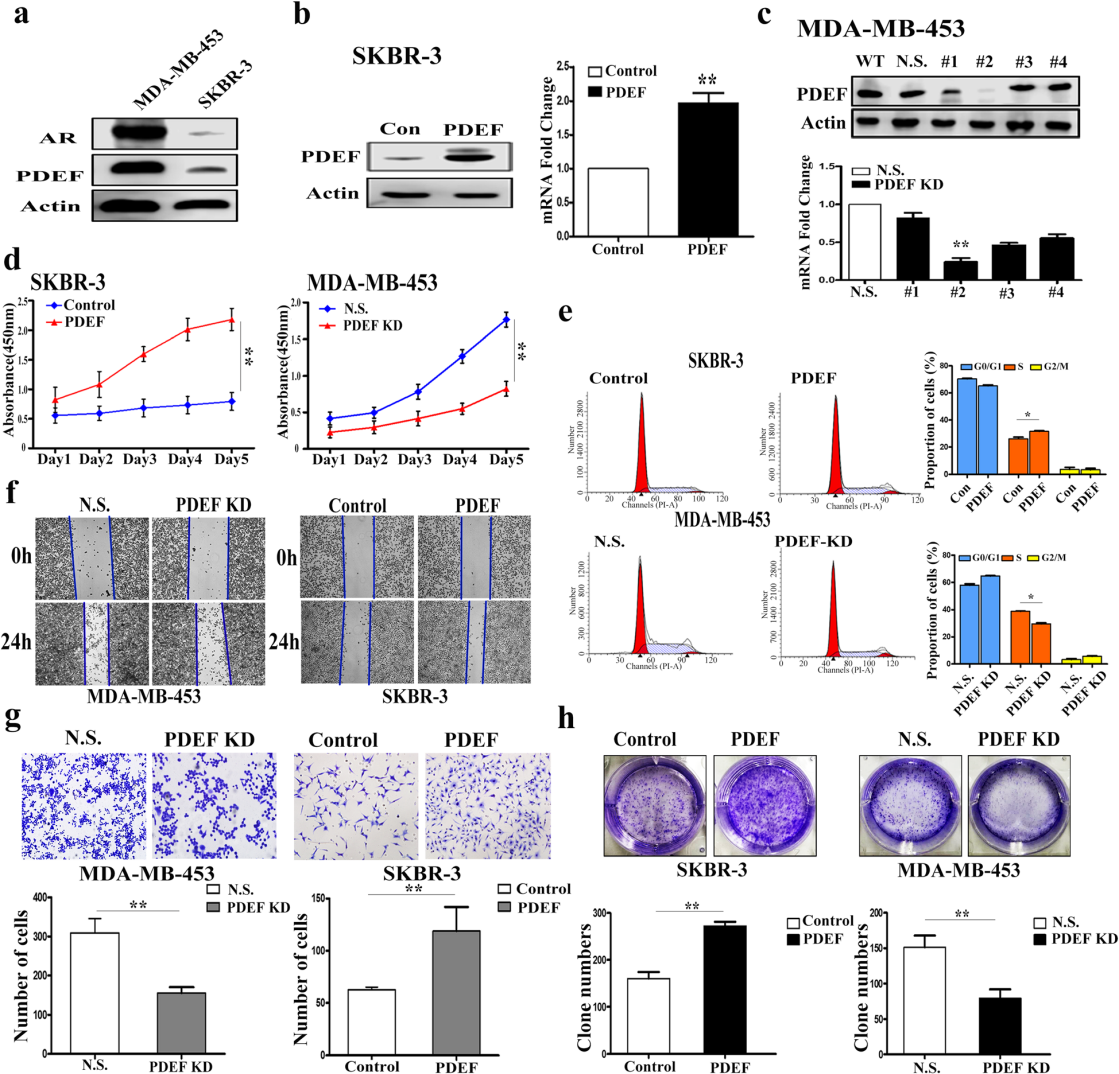
PDEF promotes the proliferation of ER-negative BC cell lines. **a** AR and PDEF protein levels in the two ER-negative BC cell lines MDA-MB-453 and SKBR-3 were determined by performing western blotting. **b** RT-qPCR and western blotting were performed to detect PDEF expression in PDEF-overexpressing SKBR-3 cells. Data are presented as mean with SD. **c** RT-qPCR and western blotting were performed to detect PDEF expression in MDA-MB-453 cells infected with the four independent PDEF-targeting shRNAs. Subsequent experiments were performed using shRNA no. #2 (WT: wild type; NS: non-specific). Data are presented as mean with SD. **d** Results of the CCK-8 assay showed that PDEF overexpression promoted SKBR-3 cell proliferation (left) and that PDEF downregulation inhibited MDA-MB-453 cell proliferation (right); **P < 0.05. **e** Results of the flow cytometry analysis showing a significant increase in the number of S-phase cells among PDEF-overexpressing SKBR-3 cells and a significant decrease in the number of S-phase cells among PDEF-downregulated MDA-MB-453 cells; **P < 0.05. **f** and **g** Wound-healing (above) and Transwell (under) assays were performed to detect the invasion and migration potential of PDEF-overexpressing SKBR-3 cells or PDEF-downregulated MDA-MB-453 cells (wound-healing assay: original magnification, × 100; Transwell assay: original magnification, × 200); **P < 0.05. **h** Colony-forming assay was conducted to determine the clone-initiating ability of PDEF-overexpressing SKBR-3 cells or PDEF-downregulated MDA-MB-453 cells; **P < 0.05

We previously analysed PDEF protein expression and clinical outcome data in the 100 ER-negative BC tissues and found a significant correlation between high PDEF expression and poor OS. These findings strongly supported the potential role of PDEF in ER-negative BC tumorigenesis. Next, we evaluated AR and PDEF protein levels in the two ER-negative BC cell lines MDA-MB-453 and SKBR-3. Both AR and PDEF proteins were highly expressed in MDA-MB-453 cells but showed low expression in SKBR-3 cells (Fig. 3a). To determine whether PDEF expression affected cellular growth, SKBR-3 cells were infected with a PDEF-expressing lentiviral vector to promote PDEF expression and MDA-MB-453 cells were infected with a PDEF-shRNA-expressing lentiviral vector to inhibit PDEF expression (Fig. 3b and c). Results of the CCK-8 assay showed increased proliferation of PDEF-upregulated SKBR-3 cells and decreased proliferation of PDEF-downregulated MDA-MB-453 cells (Fig. 3d). Results of the cell cycle analysis showed that the percentage of S-phase cells was higher among PDEF-upregulated SKBR-3 cells than among control SKBR-3 cells but was lower among PDEF-downregulated MDA-MB-453 cells than among scramble shRNA-expressing MDA-MB-453 cells (Fig. 3e). To further examine the effect of PDEF in BC tumorigenesis, we examined the biological effect of PDEF on cancer cell invasion and migration by performing cell migration and invasion assays. Cellular PDEF overexpression significantly increased the invasion and migration of SKBR-3 cells, whereas *PDEF* knockdown decreased the invasion, migration and proliferation of MDA-MB-453 cells (Fig. 3f, g and h; Additional file 1: Figure S2a and b). Together, these results suggest that PDEF promotes ER-negative BC cell proliferation.

### PDEF upregulates MYC-mediated gene transcription by promoting MAD1 degradation

**Fig. 4:**
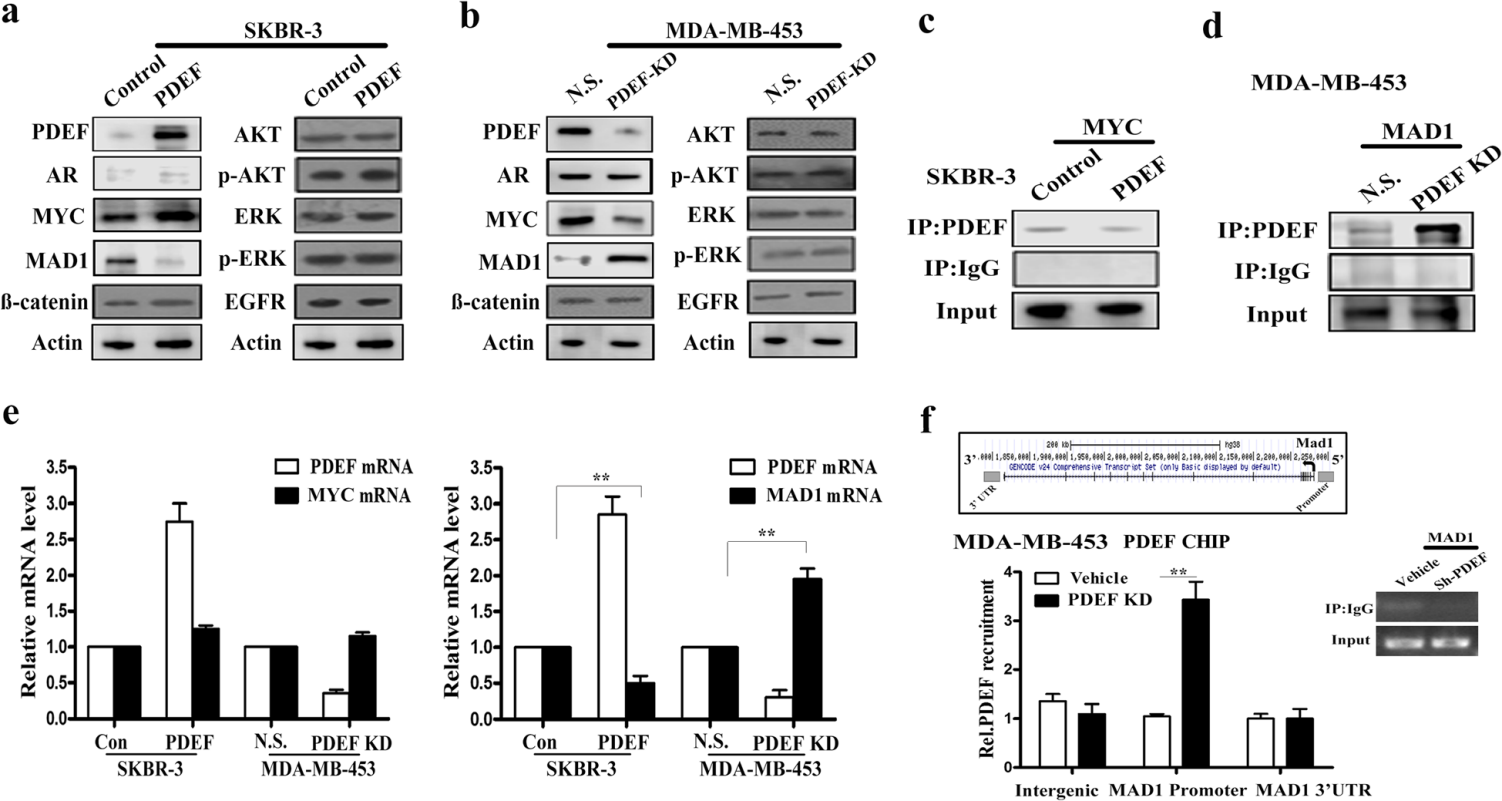
PDEF upregulates MYC-mediated gene transcription by promoting MAD1 degradation. **a** Western blotting was performed to detect the expression levels of intracellular signalling components, including MEK/ERK, PI3K/AKT, MYC/MAD1, AR and EGFR, in PDEF-overexpressing SKBR-3 cells. **b** Western blotting was performed to detect the expression levels of MEK/ERK, PI3K/AKT, MYC/MAD1, AR and EGFR in PDEF-downregulated MDA-MB-453 cells (NS: non-specific; KD: knockdown). **c** Co-IP assay was performed with the anti-PDEF antibody in PDEF-overexpressing SKBR-3 cells and control vector-infected cells. The interaction between precipitated MYC and PDEF was detected using the anti-PDEF antibody. **d** Co-IP assay was performed with the anti-PDEF antibody in PDEF-downregulated MDA-MB-453 cells and control vector-infected cells. The interaction between precipitated MAD1 and PDEF was detected using the anti-PDEF antibody. **e** *PDEF* and *MYC* mRNA levels in PDEF-overexpressing SKBR-3 cells or PDEF-downregulated MDA-MB-453 cells were determined by performing RT-qPCR (left panels). *PDEF* and *MAD1* mRNA levels in PDEF-overexpressing SKBR-3 cells or PDEF-downregulated MDA-MB-453 cells were determined by performing RT-qPCR (right panels); **P < 0.05. **f** Top panel: Schematic diagram of the PDEF-binding regions within the *MAD1* locus. Lower left panel: Results of the direct PDEF ChIP assay followed by RT-qPCR of PDEF-downregulated MDA-MB-453 cells (black bars) or control vector-infected (white bars) cells; data are presented as mean ± SD. Lower right panel: Semi-quantitative PCR of a negative control (IgG) sample

Studies have shown that AR facilitates the growth of ER-negative and AR-positive BC cells. In the present study, we found that *PDEF* was the downstream target gene of AR and that its expression was upregulated by AR. Moreover, we found that PDEF overexpression promoted the growth of ER-negative BC cells. To determine the molecular mechanism underlying the oncogenic effect of PDEF on ER-negative BC cell proliferation, we analysed AR downstream intracellular signalling components, including MEK/ERK, PI3K/AKT and MYC/MAD1. PDEF overexpression or downregulation in SKBR-3 or MDA-MB-453 cells, respectively, did not alter the expression of AKT, ERK, p-AKT and p-ERK, the key signal transducers acting downstream of AR (Fig. 4a and b). Moreover, we observed that PDEF overexpression or downregulation in SKBR-3 or MDA-MB-453 cells, respectively, did not affect AR expression, indicating the absence of a reciprocal regulatory loop between PDEF and AR. However, we found that MYC expression was significantly upregulated in PDEF-overexpressing SKBR-3 cells and was downregulated in PDEF-downregulated MDA-MB-453 cells, indicating a positive role of PDEF in regulating MYC expression. Moreover, we found that the expression of MAD1, a transcriptional repressor of MYC, was inhibited in PDEF-overexpressing SKBR-3 cells and was upregulated in PDEF-downregulated MDA-MB-453 cells, indicating a negative role of PDEF in regulating MAD1 expression.

PDEF co-immunoprecipitated with MYC in PDEF-upregulated SKBR-3 cells, which are suggested to show no interaction between PDEF and MYC (Fig. 4c). MAD1 functioned as an antagonist of MYC, and MAD1 overexpression in SKBR-3 and MDA-MB-453 cells downregulated MYC expression (Additional file 1: Figure S3a). Next, we examined whether PDEF upregulated MYC expression by promoting MAD1 degradation. We observed that PDEF co-immunoprecipitated with MAD1 in PDEF-downregulated MDA-MB-453 cells, which are suggested to show a negative interaction between PDEF and MAD1 (Fig. 4d). In addition, PDEF overexpression or downregulation in SKBR-3 or MDA-MB-453 cells, respectively, did not affect *MYC* mRNA levels (Fig. 4e), thus confirming that PDEF does not regulate MYC expression. However, PDEF upregulation in SKBR-3 cells decreased in *MAD1* mRNA expression, whereas PDEF downregulation in MDA-MB-453 cells increased *MAD1* mRNA expression, thus confirming that MAD1 is a direct target of PDEF. Results of a direct ChIP-qPCR assay showed PDEF recruitment at the *MAD1* promoter (Fig. 4f). These data suggest that PDEF upregulates MYC-mediated gene transcription by promoting MAD1 degradation.

### MAD1 suppresses PDEF-mediated growth of ER-negative BC cell lines

**Fig. 5:**
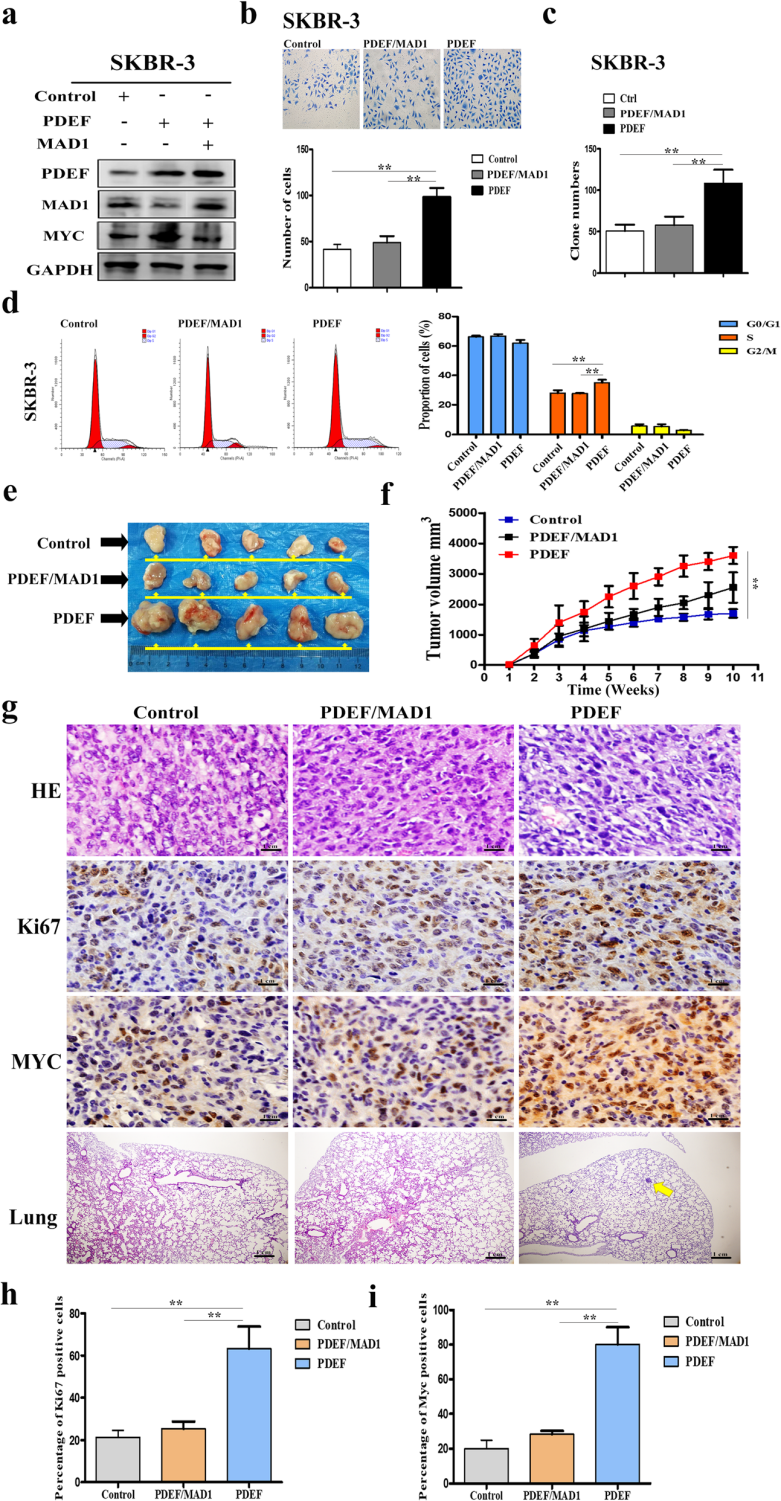
Upregulation of MAD1 expression suppresses PDEF-mediated growth of ER-negative BC cell lines. **a** PDEF, MAD1 and MYC protein levels in PDEF-overexpressing, simultaneous PDEF- and MAD1-expressing and control SKBR-3 cell clones were determined by performing western blotting. **b** Transwell assay was performed to detect the migration of the above three SKBR-3 cell clones. (Transwell assay: original magnification, × 200); **P < 0.05. **c** Colony-forming assay was performed to determine the clone-initiating ability of the above three SKBR-3 cell clones; **P < 0.05. **d** Results of the flow cytometry analysis showed that MAD1 overexpression inhibited PDEF overexpression-induced increase in the number of S-phase SKBR-3 cells; **P < 0.05. **e** Images of tumours removed from the nude mice subcutaneously injected with control SKBR-3 cells (control), stable PDEF-overexpressing SKBR-3 cell clones (PDEF) and stable simultaneous PDEF- and MAD1-overexpressing SKBR-3 cell clones (PDEF/MAD1). **f** Representative tumour growth curves for the three groups. Data are presented as mean ± SD; **P < 0.05. **g** H&E staining of tumours removed from the mice in the three groups; magnification, × 400. IHC staining of Ki67 and MYC in tumour samples obtained from the mice in the three groups (Ki67 and MYC) (magnification, × 400). Representative images of H&E staining of metastatic nodules in the lung tissues of nude mice (lungs) (magnification, × 200). **h** and **i** Statistics of the percentage of Ki67- and MYC-positive cells in the three groups; **P < 0.05

Because PDEF represses MAD1 expression, upregulation of MAD1 expression may suppress PDEF-mediated growth of ER-negative BC cells. To verify this hypothesis, we analysed PDEF-upregulated, simultaneous PDEF- and MAD1-upregulated and control SKBR-3 cells. We first confirmed MYC protein expression in SKBR-3 cells by performing western blotting. PDEF overexpression increased MYC expression; however, MAD1 expression suppressed PDEF overexpression-induced increase in MYC expression (Fig. 5a). Next, we performed the Transwell and colony-forming assays to evaluate the alteration in the invasive and stem-like properties of tumour cells. MAD1 expression suppressed PDEF overexpression-induced migration and proliferation of SKBR-3 cells (Fig. 5b and c). Results of the cell cycle analysis showed that MAD1 overexpression decreased PDEF upregulation-induced increase in the number of S-phase cells (Fig. 5d). To investigate the effect of MAD1 overexpression on PDEF, female nude mice were inoculated with stable PDEF-upregulated, stable simultaneous PDEF- and MAD1-upregulated and control SKBR-3 cell clones. We found that MAD1 over expression dramatically inhibited PDEF-mediated growth of (Fig. 5e and f) and reduced PDEF overexpression-induced Ki67 and MYC expression in (Fig. 5g, h and i) tumours isolated from the BC cell-inoculated nude mice. Moreover, compared with the mice inoculated with PDEF-overexpressing cells, the mice inoculated with MAD1-overexpressing cells did not show pulmonary metastasis. These findings indicate that upregulation of MAD1 expression can inhibit PDEF-induced proliferation of ER-negative BC cells.

### Simultaneous inhibition of AR and PDEF expression further suppresses tumour proliferation compared with the inhibition of AR alone

**Fig. 6:**
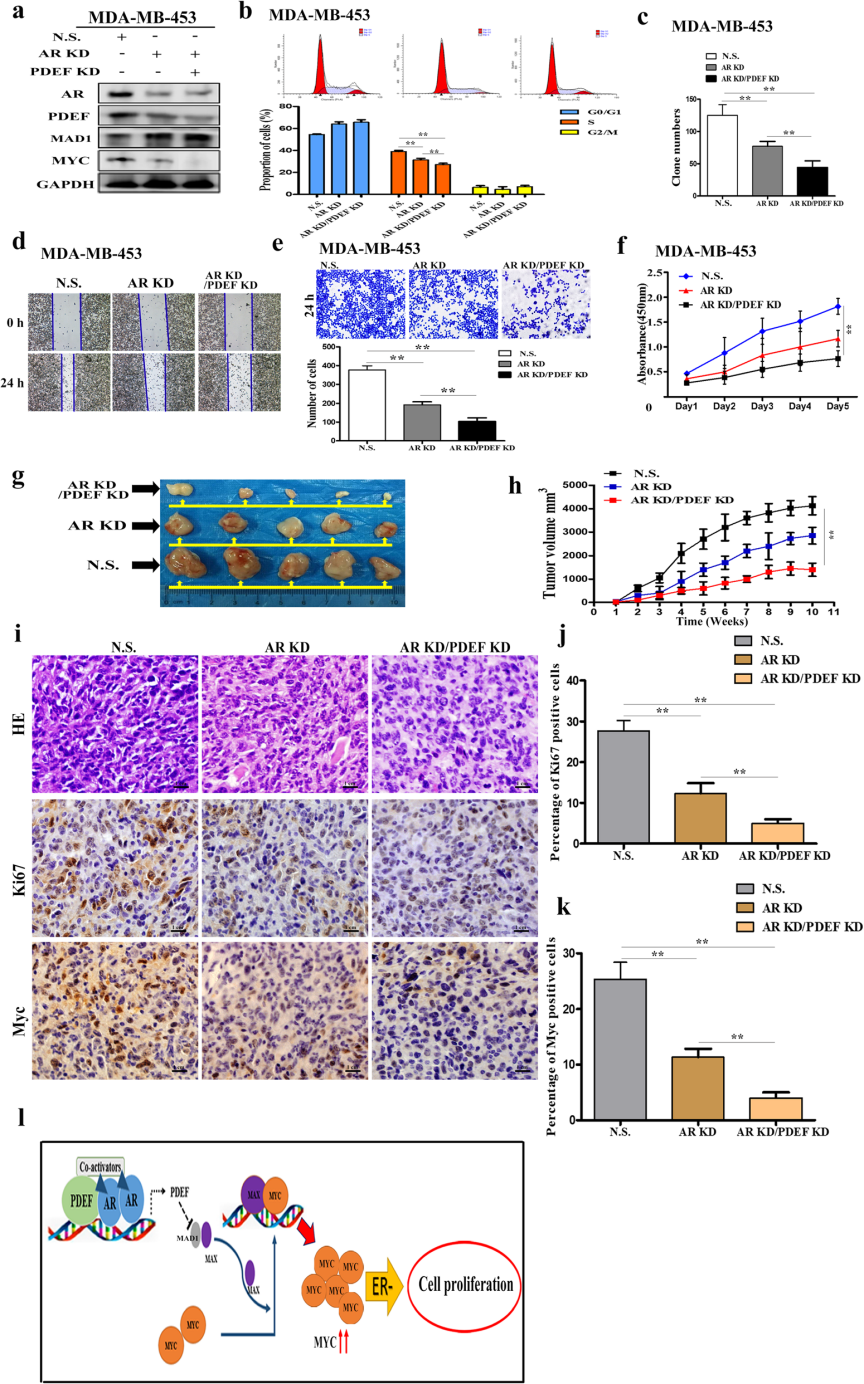
Simultaneous inhibition of AR and PDEF expression further suppresses tumour cell proliferation compared with the inhibition of AR alone. **a** AR, PDEF, MAD1 and MYC protein levels in only AR-downregulated (AR KD), simultaneous AR- and PDEF-downregulated (AR KD/PDEF KD) and control MDA-MB-453 cells (NS) were determined by performing western blotting (KD: knockdown; NS: non-specific). **b** Flow cytometry analysis was performed to detect the proliferation of the above three MDA-MB-453 cell clones; **P < 0.05. **c** Colony-forming assay was conducted to determine the clone-initiating ability of the above three MDA-MB-453 cell clones; **P < 0.05. **d** and **e** Wound-healing (left) and Transwell (right) assays were performed to detect the invasion and migration potential of the above three MDA-MB-453 cell clones (wound-healing assay: original magnification, × 100; Transwell assay: original magnification, × 200); **P < 0.05. **f** CCK-8 assay was performed to detect the proliferation of the above three MDA-MB-453 cell clones; **P < 0.05. **g** Images of tumours removed from the nude mice subcutaneously injected with control (NS), stable AR-shRNA-expressing (AR KD) and simultaneous AR-shRNA- and PDEF-shRNA-expressing stable MDA-MB-453 cell clones (AR KD/PDEF KD). **h** Representative tumour growth curves for the three groups. Data are presented as mean ± SD; **P < 0.05. **i** H&E staining of tumours removed from the mice in the three groups (magnification, × 400). Results of the IHC staining for detecting Ki67 and MYC expression in tumour samples obtained from the mice in the three groups (Ki67 and MYC; magnification, × 400). **j** and **k** Statistics of the percentage of Ki67- and MYC-positive cells in the tumour samples removed from the mice in the three groups; **P < 0.05. **l** A model showing the role of the AR–PDEF and MAD1–MYC pathways in ER-negative BC cell proliferation

We found that the AR–PDEF pathway promoted the growth of ER-negative BC cells and that *PDEF* was the downstream target gene of AR and was upregulated by AR. In addition, we found that PDEF promoted tumour proliferation and upregulated MYC-mediated gene transcription by promoting MAD1 degradation. Next, we investigated whether simultaneous inhibition of AR and PDEF expression further suppressed tumour proliferation compared with the inhibition of AR alone. For this, we analysed AR-downregulated (AR-deprived), simultaneous AR- and PDEF-downregulated (AR-/PDEF-deprived) and control MDA-MB-453 cell clones. MAD1 protein levels were higher in AR-/PDEF-deprived MDA-MB-453 cells than in AR-deprived and control MDA-MB-453 cells, whereas, MYC protein levels were lower in AR-/PDEF-deprived MDA-MB-453 cells than in AR-deprived and control MDA-MB-453 cells (Fig. 6a). Next, we examined functionally relevant changes in these cells. Results of the cell cycle analysis showed that the percentage of S-phase cells was lower among AR-/PDEF-deprived MDA-MB-453 cells than among AR-deprived and control MDA-MB-453 cells (Fig. 6b). Results of the colony-forming and CCK-8 assays showed that the proliferation of AR-/PDEF-deprived MDA-MB-453 cells was lower than that of AR-deprived and control MDA-MB-453 cells (Fig. 6c and f). Results of the wound-healing and Transwell assays showed that AR-/PDEF-deprived MDA-MB-453 cells showed significantly suppressed migration and invasion potential compared with AR-deprived and control MDA-MB-453 cells (Fig. 6d and e; Additional file 1: Figure S4a). To examine whether simultaneous inhibition of AR and PDEF expression was sufficient for inhibiting tumour proliferation and formation, female nude mice were inoculated with stable AR-downregulated, stable simultaneous AR- and PDEF-downregulated and control MDA-MB-453 cell clones. We found that the simultaneous inhibition of AR and PDEF expression dramatically inhibited tumour growth (Fig. 6g and h) and considerably reduced Ki67 and MYC expression (Fig. 6i, j and k). These results indicate that the simultaneous inhibition of AR and PDEF expression significantly suppresses the proliferation of ER-negative BC cells.

Thus, in the present study, we found that AR and PDEF are more often co-expressed and that AR directly upregulates PDEF expression, leading to its activation in ER-negative BC tissues and cells. Activated PDEF downregulates the expression of MYC transcriptional repressor MAD1, thus promoting its degradation and dissociation from MAX, the obligatory partner of MYC. In the absence of MAD1 competition, MYC forms heterodimers with MAX to sequentially induce gene transcription for promoting the proliferation of ER-negative BC cells (Fig. 6l).

## Discussion

In the present study, we identified PDEF as an oncogene and found that PDEF expression was increased in ER-negative BC tissues and was correlated with the survival of patients with ER-negative BC. Further, we found that PDEF expression was strongly correlated with AR expression in ER-negative BC cells and tissues and that *PDEF* was a direct transcriptional target of AR. Moreover, we found that PDEF upregulated oncogene *MYC* expression by downregulating MAD1 expression and promoted BC cell proliferation and metastasis both in vitro and in vivo. Simultaneous inhibition of AR and PDEF expression further suppressed ER-negative BC cell proliferation both in vitro and in vivo. Thus, our results highlight a novel mechanism of AR signalling activation in ER-negation BC and suggest that PDEF is a new potential therapeutic target for treating patients with ER-negative BC.

ER-negative breast carcinoma constitutes approximately 30% of all BC cases and commonly affects a young patient population compared with ER-positive breast carcinoma [26, 27]. Studies have shown that AR is expressed in approximately 60–70% cases of ER-negative BC. Thus, the AR signalling pathway plays a significant role in the proliferation and survival of ER-negative BC, and AR inhibition suppresses the proliferation of ER-negative and AR-positive BC cells both in vitro and in vivo [28–31].

Doane et al. performed a genome-wide expression analysis of 99 primary BC samples and eight BC cell lines and found that AR and PDEF were overexpressed in ER-negative BC tissues and cells [14]. PDEF expression is suggested to be relevant for the sub-classification of AR^+^ BC [7]. This suggests that PDEF plays an important role along with AR in ER-negative BC. In the present study, we first examined AR and PDEF expression in the 100 specimens obtained from patients with ER-negative BC by performing IHC. We found that both AR and PDEF were highly expressed and were more often co-expressed in ER-negative BC tissues. The results of survival analysis showed that PDEF overexpression as well as AR and PDEF co-expression were associated with the poor OS of patients with ER-negative BC. Furthermore, analysis of *PDEF* mRNA and protein levels in the two ER-negative BC cell lines MDA-MB-453 and SKBR-3 indicated that *PDEF* was a downstream target gene of AR and was upregulated by AR. These results confirm the close relationship between AR and PDEF and the critical function of PDEF as a specific regulator of ER-negative BC cell survival.

Studies assessing PDEF function in different cancers suggest its important role in tumorigenesis [32, 33]. Studies on BC have shown that PDEF promotes the luminal differentiation of basal mammary epithelial cells and contributes to endocrine resistance in ER-positive BC [13]. In contrast, other studies have shown that PDEF levels decrease in highly malignant, ER-negative and basal-like BC cells and that re-expression of PDEF in these cells reduces their migration and invasion, suggesting that PDEF functions as a tumour suppressor [33, 34]. It is difficult to assess the relevance of ectopic PDEF expression in tumour cell lineages. We speculated that luminal epithelial cell-specific transcription factors such as PDEF reduced the epithelial properties of these cells by increasing their invasive and migratory potential because we found that both AR and PDEF were highly expressed and were more often co-expressed in these cells. In the present study, we found that AR and PDEF protein levels were high in MDA-MB-453 cells and were low in SKBR-3 cells. To examine the role of PDEF in ER-negative BC cells, high PDEF-expressing MDA-MB-453 cells were infected with a PDEF-shRNA-expressing lentiviral vector to inhibit PDEF expression and low PDEF-expressing SKBR-3 cells were infected with a PDEF-expressing lentiviral vector to promote PDEF expression. The results of these gain- and loss-of-function cellular studies indicated a positive effect of PDEF expression on the growth, migration and invasion of ER-negative BC cells. These results were consistent with the results of our IHC analysis that showed that PDEF functions as an oncogenic factor in ER-negative BC.

MYC and its negative regulator MAD1 play an important role in BC progression [35–37]. We found that PDEF overexpression or downregulation altered the expression of MYC and its transcriptional repressor MAD1. PDEF positively regulated MYC expression and negatively regulated MAD1 expression. Results of the Co-IP assay showed that PDEF did not interact with MYC but interacted with the regulatory region of MAD1 in ER-negative BC cells. These results suggest that PDEF indirectly upregulates MYC expression by disrupting MAD1 expression. To validate this, we upregulated MAD1 expression in PDEF-overexpressing SKBR-3 cells and found that the upregulation of MAD1 expression significantly inhibited PDEF-induced proliferation and invasion of these cells. Thus, our results indicate that PDEF upregulates oncogene *MYC* expression by downregulating MAD1 expression and promotes BC cell proliferation a both in vitro and in vivo. Moreover, our results highlight the AR–PDEF–MAD1–MYC axis and provide a novel mechanism of the AR signalling pathway associated with the proliferation of ER-negative BC cells.

Because AR maintains the proliferation of ER-negative BC cells, the use of AR antagonists seems to be a logical choice for treating this cancer subtype [38–40]. Many studies have suggested that bicalutamide and enzalutamide, which are non-steroidal anti-androgens, competitively inhibit the binding of androgens to AR in ER-negative BC [41, 42]. Our results indicate that *PDEF* is involved in the proliferation and invasion of ER-negative BC cells and is a direct transcriptional target of AR. Moreover, our results suggest that PDEF inhibition has a therapeutic value for treating ER-negative BC. Our results also indicate that simultaneous suppression of AR and PDEF expression further suppresses tumour proliferation both in vitro and in vivo compared with the inhibition of AR expression alone. These results suggest that PDEF is not only an essential factor in the AR-associated transcriptional network but also a potential therapeutic target for treating patients with ER-negative breast carcinoma.

## Conclusions

In summary, we found that PDEF functions as an oncogene in ER-negative BC and is an independent predictor of the survival of patients with this cancer subtype. PDEF is an AR-associated factor and is positively regulated by AR. Moreover, PDEF upregulates MYC-mediated gene transcription by promoting MAD1 degradation. Furthermore, the AR–PDEF signalling pathway promotes ER-negative BC cell proliferation, suggesting that PDEF is a new therapeutic target for treating ER-negative BC.

## Additional file

Additional file 1: **Figure S1.** PDEF is directly regulated by AR in SKBR-3 cells. **Figure S2.** PDEF promotes the migration of ER-negative BC cells. **Figure S3.** MAD1 functions as a negative regulator of MYC. **Figure S4.** Simultaneous inhibition of AR and PDEF expression further suppresses tumour migration compared with the inhibition of AR alone. (DOCX 755 kb)
