# Supplementary material for: AR–PDEF pathway promotes tumour proliferation and upregulates MYC-mediated gene transcription by promoting MAD1 degradation in ER-negative breast cancer
Source: Mol Cancer. 2018 Sep 14;17:136. doi: 10.1186/s12943-018-0883-0 (PMC6138935; doi:10.1186/s12943-018-0883-0)
Supplement: Supplementary file 1 — Figure S1. PDEF is directly regulated by AR in SKBR-3 cells. Figure S2. PDEF promotes the migration of ER-negative BC cells. Figure S3. MAD1 functions as a negative regulator of MYC. Figure S4. Simultaneous inhibition of AR and PDEF expression further suppresses tumour migration compared with the inhibition of AR alone. (DOCX 755 kb) [file 12943_2018_883_MOESM1_ESM.docx]

**Supplemental data**

**Supplementary shRNAs, qPCR Primers and ChIP Primers**

**Expression shRNAs (Target Sequence):**

**PDEF shRNAs:**

Sh-PDEF#1: 5’-TGCTCAACATCACCGCAGATC-3’

Sh-PDEF#2: 5’-TTCATTAGGTGGCTCAACAAG-3’

Sh-PDEF#3: 5’-TCCCGCCATGAACTACGACAA-3’

Sh-PDEF#4: 5’-TGAAGTTGGCACTGCAGCAGA-3’

**AR shRNAs:**

Sh-AR#1: 5’-GCAGGATCGAGTTATTGTTAA-3’

Sh-AR#2: 5’-CCGCTGACCTTAAAGACATCC-3’

Sh-AR#3: 5’-CCCATTGACTATTACTTTCCA-3’

Sh-AR#4: 5’-GCACTATTGATAAATTCCGAA-3’

**Vector shRNA Target Sequences:**

5’-GCTTCGCGCCGTAGTCTTA-3’

**Expression qPCR Primers:**

**AR:**

Forward, 5’-TTTGCCCATTGACTATTACTTTCC-3’

Reverse, 5′-TTTCCCTTCAGCGGCTCTTT-3’

**PDEF:**

Forward, 5’-GTGGCTCAACAAGGAGAAGG-3’

Reverse, 5’-GTGCACGAACTGGTAGACGA-3’

**MYC:**

Forward, 5’-ATGCCCCTCAACGTTAGC-3’

Reverse, 5′-AGCTCGCTCTGCTGCTGC-3′

**MAD1:**

Forward, 5’-GTGTGGGCCCGGTTTCCCCTC-3’

Reverse, 5’-CTGGCAGTGTGACACAGTCCA-3’

**GAPDH:**

Forward, 5’-CGAGATCCCTCCAAAATCAA-3’

Reverse, 5’-TTCACACCCATGACGAACAT-3’

**ChIP Primers:**

**AR-ChIP-PDEF Promoter:**

Forward, 5’-CCTCACCTGTTCCTTCTTGC-3’

Reverse, 5’-CCCTGAAACCGTTAGAGCAG-3’

**AR-ChIP-Enhancer 1, PDEF gene:**

Forward, 5’-GTCTGGAGGCCCCTTTCTAC-3’

Reverse, 5’-AAACTTTGCACAAGCCGAGT-3’

**AR-ChIP-Enhancer 2, PDEF gene:**

Forward, 5’-GCAGGAACACATTGCATCAG-3’

Reverse, 5’-CTTGTCCGTCAAGTGGGAAT-3’

**AR-ChIP-PDEF 3’ UTR:**

Forward, 5’-TGTCTGCAGGGACTCAGTTG-3’

Reverse, 5’-TGGCAGTGAGCAAATAGCAC-3’

**PDEF-ChIP-MAD1 Promoter:**

Forward, 5’-GTGTGGGCCCGGTTTCCCCTC-3’

Reverse, 5’-CTGGCAGTGTGACACAGTCCA-3’

**PDEF-ChIP-MAD1 3’ UTR:**

Forward, 5’-ACTCTGCACTGCCAGACAAA-3’

Reverse, 5’-TGGAAACCACATTTTGGTCA-3’

**Intergenic Negative Controls:**

Forward, 5’-TTCACTCCCATTACCCAAGC-3’

Reverse, 5’-CCCAGCTACTCAGGAGGATG-3’


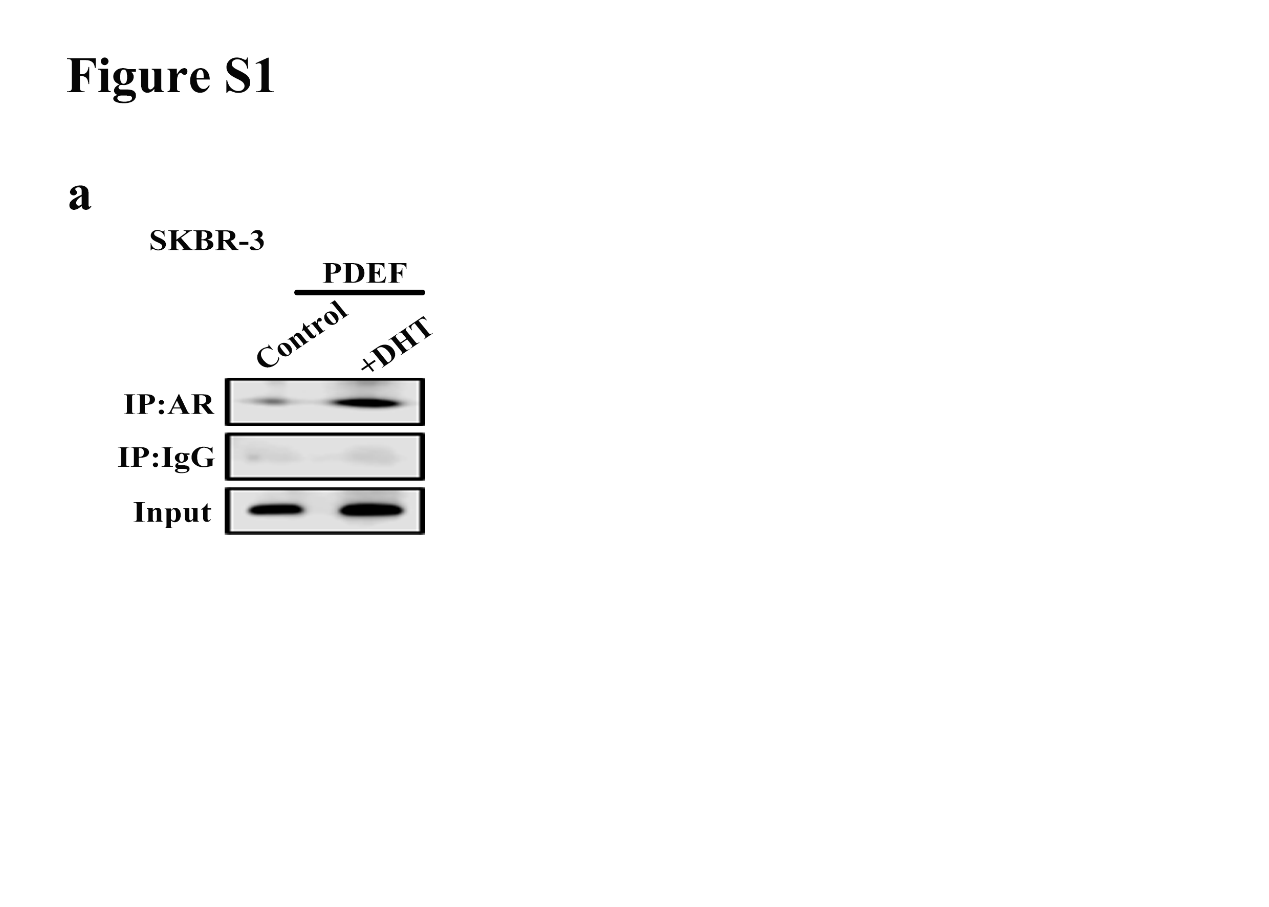


**Figure S1. PDEF is directly regulated by AR in SKBR-3 cells.** (a) Co-IP assay was performed with the anti-AR antibody in SKBR-3 cells treated with 1 nM DHT for 48 h or infected with a control vector. The interaction between the precipitated PDEF and AR was detected using the anti-AR antibody.





**Figure S2. PDEF promotes the migration of ER-negative BC cells.** (a and b) Wound-healing assay was performed to detect the invasion potential of PDEF-downregulated MDA-MB-453 cells or PDEF-overexpressing SKBR-3 cells (magnification, ×100).


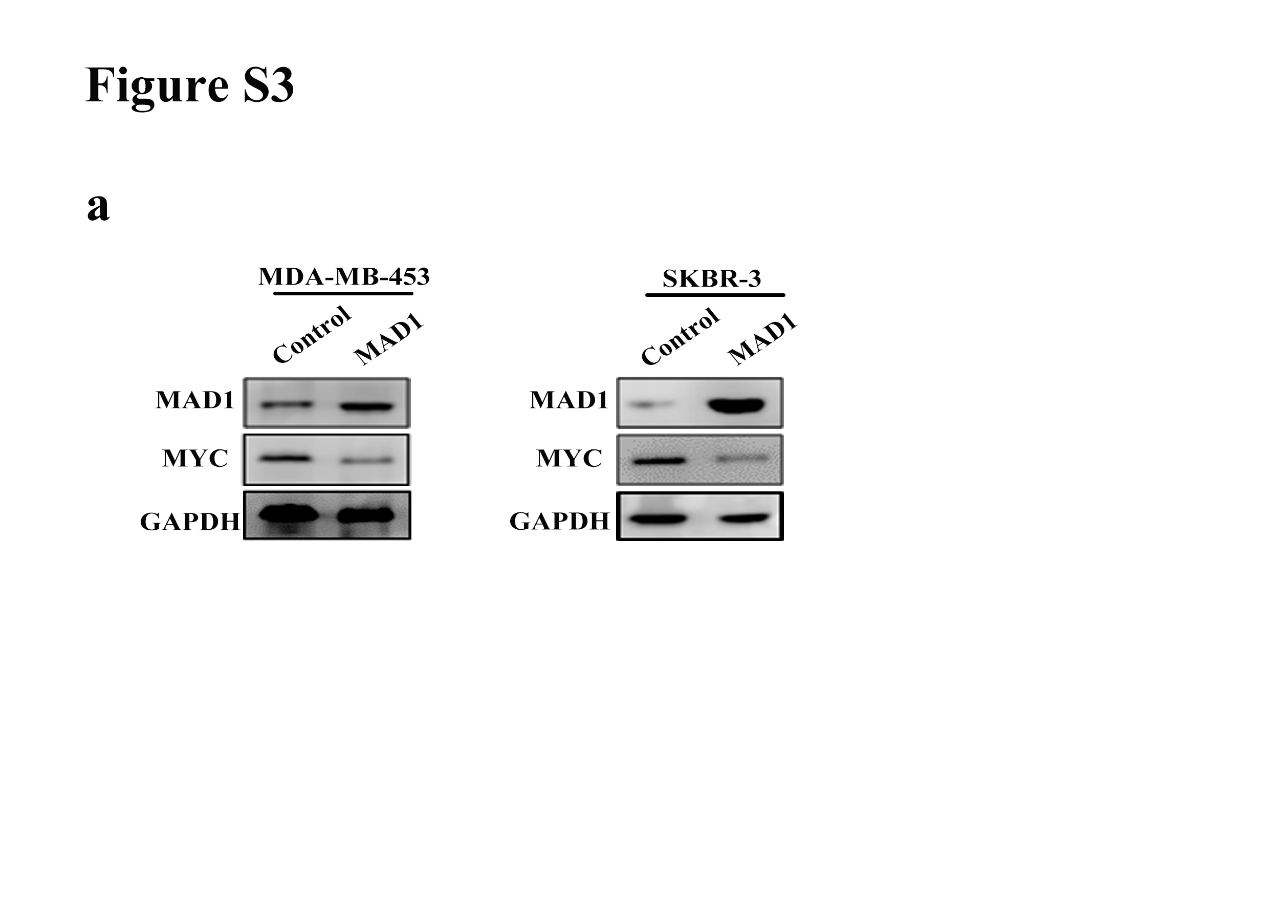


**Figure S3. MAD1 functions as a negative regulator of MYC.** (a) Western blotting was performed to detect MYC expression in MAD1-overexpressing MDA-MB-453 and SKBR-3 cells.





**Figure S4. Simultaneous inhibition of AR and PDEF expression further suppresses tumour migration compared with the inhibition of AR alone.** (a) The wound-healing assay was performed to detect the invasion potential of AR-downregulated (AR KD), simultaneous AR- and PDEF-downregulated (AR KD/PDEF KD) and control (NS) MDA-MB-453 cell clones (magnification, ×100) (KD: knockdown; NS: non-specific).
